# iMOSS: an integrated open-source tail suspension test platform for high-resolution immobility scoring and synchronization with neural activity

**DOI:** 10.3389/fnbeh.2026.1819512

**Published:** 2026-05-08

**Authors:** Zengyou Ye, Xia Min, Sarah T. Johnson, Xuehong Cao, Satoshi Ikemoto

**Affiliations:** 1 Behavioral Neuroscience Research Branch, Intramural Research Program, National Institute on Drug Abuse, National Institutes of Health, Baltimore, MD, United States; 2 Molecular Cardiology Research Institute, Tufts Medical Center, Boston, MA, United States

**Keywords:** behavioral neuroscience, fiber photometry, immobility scoring, open-source platform, tail suspension test

## Abstract

The tail suspension test (TST) is widely used to assess stress-coping behavior in rodents, characterized by alternating periods of active (struggling) and passive (immobile) responses. Immobility in the TST is interpreted as behavioral despair and serves as a key measure for screening antidepressant compounds. Traditional manual scoring is labor-intensive and temporally imprecise, while existing automated systems often misclassify behaviors and have not shown the capacity to integrate behavioral data with neural recording methods. Here, we improved upon the traditional TST with our new developed iMOSS (Immobility/Mobility Optimized Scoring System)—two open-source, low-cost, and scalable tools for high-resolution quantification of mobility and immobility: (1) iMOSS-MV, a video-based frame-by-frame manual-scoring software instrument designed to precisely annotate the exact onset frame for each binary event, and (2) iMOSS-AS, a sensor-based automated instrument detecting immobility/mobility bouts from the sensor-signal using a machine-learning optimized detection threshold. The output from iMOSS-AS closely matched that from iMOSS-MV and outperformed other publicly available tools. Moreover, both systems reliably detected changes in mobility and immobility induced by imipramine treatment, demonstrating sensitivity to pharmacological manipulation. Finally, both iMOSS tools readily integrated with neural data, as shown by simultaneous analysis of medial septal glutamatergic calcium activity via fiber photometry. Thus, either iMOSS-MV or iMOSS-AS alone offers an efficient, user-friendly, and bias-minimized platform for high-throughput behavioral analysis, enabling seamless integration of behavioral and neural data in systems neuroscience research.

## Introduction

The tail suspension test (TST) is widely used to assess coping responses in rodents, characterized by alternating active (struggling) and passive (immobile) periods. Immobility in the TST is interpreted as behavioral despair, which indexes antidepressant efficacy. In addition to its role in drug screening, the TST provides a powerful assay for probing neural mechanisms underlying active and passive coping strategies during inescapable stress. Traditionally, TST behavior has been scored manually using general video playback software (e.g., Can et al., 2012), which remains the preferred approach for accuracy but has notable limitations such as labor intensiveness, variability between observers, and systematic temporal offset annotation from the true onset frame, compounded by the lack of dedicated software that supports frame-accurate and efficient behavior annotation. Although it is not possible to address all limitations, developing tools that enable frame-by-frame annotation would greatly improve temporal precision and support direct integration of behavioral scores with neurophysiological measurements.

Several automated video-based tools have been developed (Juszczak et al., 2006; Meng et al., 2024; Nandi et al., 2021). These tools typically quantify activity by detecting changes in grayscale intensity or silhouette area across frames. For example, DBscorer (Nandi et al., 2021) and EthoVision^®^ XT (Noldus) classify mobility and immobility by measuring frame-to-frame differences in binarized image area. However, such methods frequently misclassify partially occluded movements (e.g., isolated limb motion hidden behind the body) as immobility states, while true immobility (e.g., passive swinging or trunk rotation) may be mislabeled as mobility. These errors are compounded by the inherent limitations of 2D video, where occlusion or unfavorable camera angles (e.g., when the back of the mouse faces the camera) obscure critical features. Consequently, video algorithms may not accurately classify behavioral states.

Sensor-based approaches offer an alternative by detecting mechanical force fluctuations rather than relying on visual cues. Systems using strain gauges or load cells improve temporal precision and reduce classification errors compared with video-based methods (Juszczak et al., 2006; Steru et al., 1987). In addition, these systems can be built at a fraction of the cost of commercial platforms.

In response to these challenges, we developed two independent instruments named iMOSS (Immobility/Mobility Optimized Scoring System), a flexible, open-source, and low-cost platform for precise immobility quantification. iMOSS-MV is a video-based manual annotation tool with frame-level resolution designed to overcome the temporal imprecision of conventional scoring, and iMOSS-AS, is a sensor-based automated hardware-software instrument with 80 Hz resolution and machine learning-based detection threshold optimization. A key feature of iMOSS is its robust integration with modern neuroscience tools through two complementary modalities. For hardware integrated directly within the Bonsai workflow (e.g., Neurophotometrics systems), a shared software clock enables seamless, high-precision temporal alignment. For standalone external hardware, the system supports bidirectional TTL communication. It can send TTL pulses to be recorded as digital time markers by external systems (e.g., Doric photometry systems) or receive TTL pulses from such systems to achieve precise timestamp alignment. This platform supports simultaneous testing of up to four mice, includes a dedicated platform for secure positioning and neural interface connections. These features make iMOSS-AS a versatile solution that addresses the limitations of existing methods for behavioral phenotyping and integrative neuroscience research.

In practice, either iMOSS-MV or iMOSS-AS alone is sufficient to assess ongoing coping responses with moment-by-moment precision. In this paper, we will discuss iMOSS-MV first and establish its validity in assessing coping responses, and then iMOSS-MV is used as the reference standard to validate iMOSS-AS.

## Materials, equipment, and methods

### Code accessibility

The iMOSS-MV and iMOSS-AS were developed in Python and leverages widely used libraries including OpenCV, Tkinter, Numpy, Pandas, Pillow, Scipy, and PyExcelerate. All the codes will be freely available online at https://github.com/addy9908/iMOSS under an MIT license, along with user documentation detailing the installation process. Additionally, the full Python source code is provided in the Supplementary material. Future updates to the code will be available through the link provided above.

### Animals

The C57BL/6 mice were purchased from Jackson Laboratory (Bar Harbor, ME), while transgenic vGluT2-Cre mice were obtained from Jackson Lab then bred at the National Institute on Drug Abuse Intramural Research Program animal facility. All mice were group-housed in a colony room maintained at a constant temperature of 70–74°F and relative humidity of 35–55%. Animals were kept on a 12-h light/dark cycle (lights on at 07:00 a.m.) with *ad libitum* access to food and water, except during the 6-min testing period. All procedures were approved by the Animal Care and Use Committee of Intramural Research Program of the National Institute on Drug Abuse and were conducted in accordance with the National Research Council Guide for the Care and Use of Laboratory Animals. To minimize animal use, validation of the iMOSS system was performed exclusively in male mice with diverse immobility times.

For the development, validation, and benchmarking of iMOSS-MV and iMOSS-AS tests with pharmacological manipulation, sixteen male age-matched adult C57BL/6J mice (∼3 months old with body weight at 25.47 ± 0.24 g) were used, comprising eight saline-treated and eight imipramine-treated animals (15 mg/kg, intraperitoneal injection, 30 min before tail suspension testing). All behavioral data were collected and analyzed under a double-blind protocol. Treatment identities were encoded prior to analysis, and data from all 16 mice were pooled and processed in randomized order to prevent experimenter bias. The encoded group information was decoded only after all blinded analyses were completed, to ensure unbiased algorithm training and evaluation.

To validate for neural synchronization, a separate cohort of male vGluT2-Cre [Slc17a6tm2(cre)Lowl] mice (3–4 months old) was used. These transgenic mice express Cre recombinase under the vesicular glutamate transporter 2 (vGluT2) promoter and were used for selective expression of GCaMP8s in medial septal glutamatergic neurons. Data from this cohort were used exclusively for proof-of-concept demonstrating the capability of iMOSS to synchronize behavioral scoring with neural activity recorded by a fiber-photometry procedure.

### Surgery

The vGluT2-cre mice were anesthetized with a 1.5% isoflurane-oxygen mixture and placed under a stereotaxic apparatus. An AAV-syn-FLEX-jGCaMP8s (200 nL, 9 × 10^12^ vg/m, 162377-AAV9, Addgene) was microinjected into medial septum (MS: AP 1.0, ML 0.0, DV 3.8 mm) with a syringe pump (UMP3, World Precision Instruments) at 50 nL/min, with additional 8 min waiting time before withdrawing the needle (34G, blunted). The optic fibers (NA: 0.37; core diameter: 200 μm, RWD Life Science Co., Ltd.) were then implanted into MS (0.2 mm above injection site) and secured on the skull with dental cement (Geristore A and B cement, Denmat; part #s 4506 and #034522101). After recovery, the mice were returned to the cage and monitored closely for their health status in the following 3 days, with additional > 4 weeks recovery before TST test.

### Histology

Mice were anesthetized in an induction chamber with 3–5% isoflurane until fully immobile. Under deep anesthesia, animals were euthanized by transcardial perfusion with 0.1 M phosphate-buffered saline (PBS) followed by 10% neutral-buffered formalin (NBF). Brains were post-fixed in 10% NBF overnight followed by 30% sucrose for 72 h. Coronal sections (40 μm) were cut using a cryostat (Leica CM3050 S), and viral expression as well as optical fiber placement were verified under a fluorescence microscope (Keyence BZ-X720).

### TST procedure

To acclimate animals to the experimenters, each mouse was handled for 5–10 min per day over 3 days in the testing room prior to the tail suspension test (TST). During testing, the mouse was suspended by its tail using a 12 cm strip of adhesive tape (Cole-Parmer, EW-06530-38) affixed to a hanging bar within a custom-built TST box, after taring the load cell signal before use. The test lasted 6 min and was conducted under continuous white noise (70–72 dB) to minimize external distractions. To prevent tail-climbing behavior—a common confounding factor in TST scoring—a lightweight plastic tube (40 × 10 × 8.5 mm; L × OD × ID; ∼0.9 g) was gently placed around the mouse’s tail, following established protocols (Can et al., 2012). Each animal was under continuous visual monitoring by the experimenter during the test. Pre-defined criteria were established for the early termination of the test for any animal that showed signs of excessive distress, such as labored breathing or vocalization, to ensure animal wellbeing.

After testing, the mice were promptly returned to their home cage. The entire session was recorded using a webcam positioned horizontally at the mouse’s eye level, approximately three feet from the test box. The webcam was connected to a desktop computer, and the video was captured in .avi format using Bonsai-RX (Lopes et al., 2015), an open-source visual programming language for real-time data acquisition. A frame-to-time mapping was simultaneously recorded and saved as a .csv file, providing precise timing information for each frame. Recorded videos were subsequently analyzed using the iMOSS-MV scoring software, and the processed data were visualized using GraphPad Prism (v. 10.4.2), MATLAB or Python.

### Threshold optimization using machine learning

Automated immobility thresholds were optimized using a machine learning-based prediction framework. For each mouse, automated immobility time was computed across a range of candidate thresholds with iMOSS-AS and paired with manually scored immobility times obtained by blinded observer A with iMOSS-MV. No deletion or filling was performed for data cleaning. To optimize the immobility-detection threshold for the iMOSS-AS system in a way that generalizes across animals, we used a Random Forest regression model because of its ability to capture nonlinear relationships and its robustness to overfitting. The dataset included mouse ID, candidate threshold values, total immobility time calculated using iMOSS-AS at each threshold (Auto_Time), and total immobility time obtained using iMOSS-MV (Manual_Time). Manual_Time was used as the target variable (reference standard), with Threshold and Auto_Time as input features. To prevent data leakage, data were split at the animal level (75% training, 25% test; random_state = 42), ensuring that all observations from a given mouse were confined to a single set. The held-out test set was not used for model training, hyperparameter tuning, or model selection. Model training and selection were performed on the training data using grouped cross-validation (GroupKFold, 5-folds; grouped by mouse), and performance was evaluated using mean absolute error (MAE), averaged across folds. Using scikit-learn (v1.5.1), a baseline Random Forest regressor (n_estimators = 200, random_state = 42; all other parameters set to default values) was compared with a model tuned using RandomizedSearchCV over predefined hyperparameter ranges, using the same grouped cross-validation scheme. Based on cross-validation performance, the baseline model was selected and carried forward for all subsequent analyses. The selected model was then trained on the full training dataset prior to evaluation. Final performance was evaluated once on the held-out test set using MAE, and agreement between cross-validation and test performance was assessed to evaluate potential overfitting. The model was then used to estimate both individual optimal thresholds and a single global threshold that minimizes prediction error across animals, with the global value confirmed to lie within the central range of individual thresholds.

### Evaluation of automated iMOSS-AS scoring

Because the automated scoring methods operated at different sampling resolutions, all outputs were first resampled to a common temporal grid defined by the smallest time step among the methods. Each method’s immobility time series was interpolated onto this grid using nearest-neighbor assignment to preserve the binary (immobility vs. mobility) structure of the data. Manual scoring with iMOSS-MV served as the reference standard for all comparisons.

Performance was evaluated using two complementary metrics. The F1-score was calculated as the harmonic mean of precision and recall:


$$\mathrm{F1}=\frac{2\,x\,\mathrm{Precision}\,x\,\mathrm{Recall}}{\mathrm{Precesion}+\mathrm{Recall}},\mathrm{where}$$

$P\,r\,e\,c\,i\,s\,i\,o\,n=\frac{\mathrm{TP}}{\mathrm{TP}+\mathrm{FP}}$, $R\,e\,c\,a\,l\,l=\frac{\mathrm{TP}}{\mathrm{TP}+\mathrm{FN}}$, and TP = true positives, FP = false positives, FN = false negatives relative to manual annotations.

The Cohen’s Kappa coefficient was computed to quantify agreement between automated and manual scoring while correcting for chance-level concordance: $\kappa =\frac{P_{o}-P_{e}}{1-P_{e}}$, where P_*o*_ is the observed agreement and P_*e*_ is the expected agreement by chance. This correction is particularly important given the natural imbalance between mobility and immobility states.

Together, these metrics provided a standardized and complementary evaluation of automated scoring methods relative to manual annotation.

### Fiber photometry recording and analyses

Fiber-photometry recording from the MS were performed using a Neurophotometrics FP3002 system (Neurophotometrics Ltd.) via patch cables (NA 0.37; core diameter 200 μm) coupled to cannulas implanted in vGluT2 mice during the TST. Isosbestic (415 nm LED, 50 μW) and calcium-dependent GCaMP (470 nm LED, 50 μW) fluorescence signals were sampled continuously at 40 Hz and synchronized with video via a custom Bonsai-Rx pipeline.

Signal processing was conducted in MATLAB: isosbestic and GCaMP channels were separated, and the isosbestic trace was fit to GCaMP using robust regression to compute ΔF/F as (F-F_*fitted_iso*_)/F_*fitted_iso*_, followed by Z-score normalization. Using the timestamps recorded in Bonsai-Rx, GCaMP activity and detected calcium peaks (MATLAB’s findpeaks function, MinPeakProminence = 0.5) were temporally aligned to TST immobility bouts scored with iMOSS-AS, enabling comparison of neural dynamics before and after behavioral transitions using *post hoc t*-tests. During the alignment, only event-confined segments were included in the plot and analysis. Time points extending beyond event boundaries were excluded, and averages were calculated from event-pure data only.

### Statistics

We used GraphPad Prism 10.5.0 to analyze data. Statistical results were summarized in Statistical Table (Supplementary material). Linear regression was used to assess correlations between two groups. Comparisons between two groups were performed using two-tailed unpaired Student’s *t*-tests. One-way repeated-measures ANOVAs with Dunnett’s *post-hoc* tests were used to compare F1-scores and Cohen’s Kappa coefficients among scoring methods (iMOSS-AS, DBscorer, EthoVision-2.5, and EthoVision-3). Two-way repeated-measures ANOVA xwith Šídák’s *post-hoc* test was used to compare immobility measures obtained from different scoring methods across saline- and imipramine-treated groups. Three-way repeated-measures ANOVA with Geisser-Greenhouse correction was applied to analyze cumulative immobility time across saline- and imipramine-treated groups scored with iMOSS-MV and iMOSS-AS. All data are presented as mean ± SEM unless otherwise indicated.

## Results

### Custom-designed TST apparatus

**FIGURE 1:**
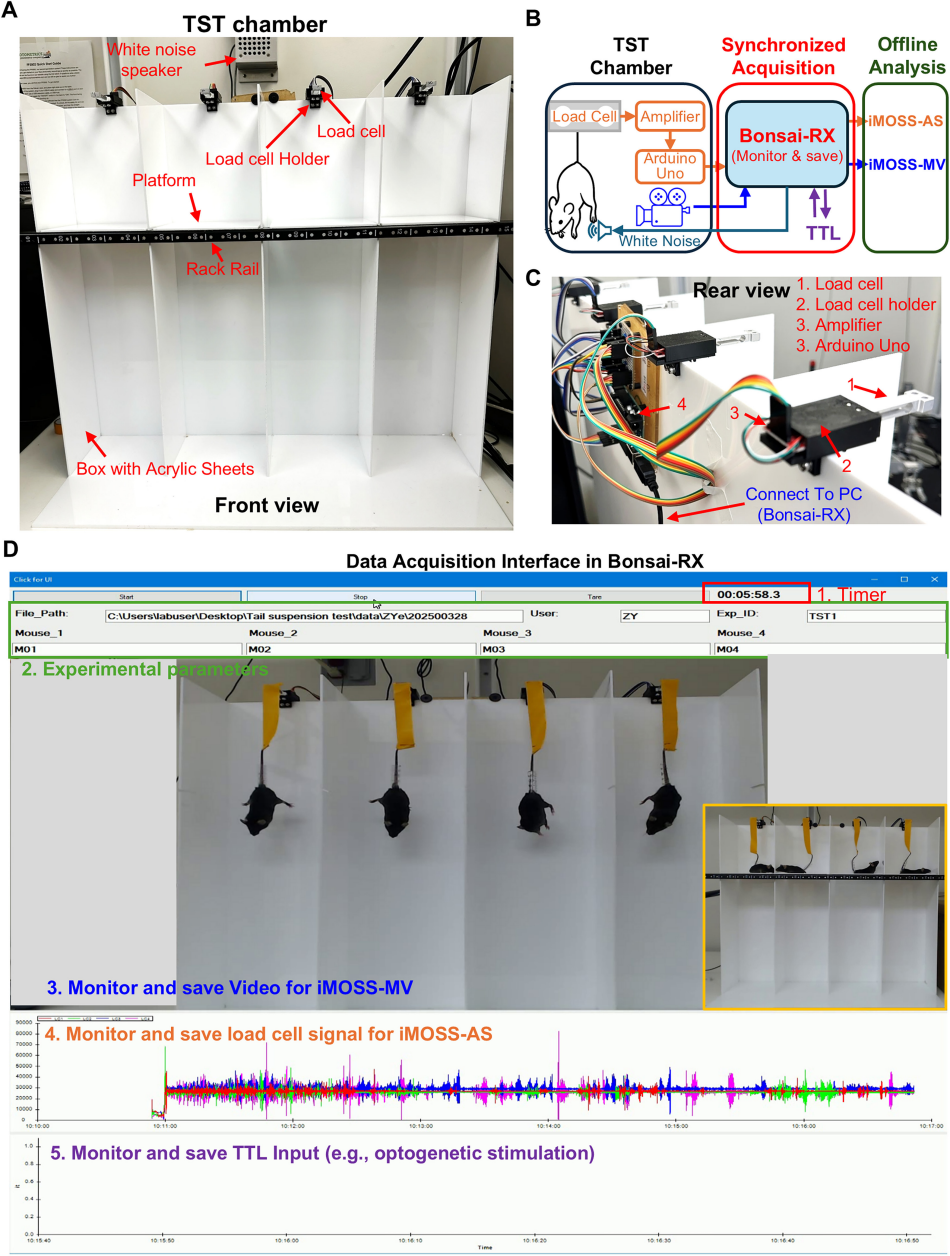
iMOSS hardware and software designs for behavioral data acquisition. **(A)** Front view of TST chamber constructed from acrylic panels equipped the resting platform. The addition of four load cells is needed for simultaneous motion detection across compartments. **(B)** Workflow chart from behavioral detection to offline analysis. **(C)** Rear top view of the TST chamber highlighting components of the iMOSS-AS hardware module, including load cells, amplifiers, and the Arduino Uno used for real-time data processing. **(D)** Graphical user interface developed in Bonsai-RX for synchronized data acquisition, displaying real-time video, load-cell signals, and TTL inputs for monitoring and saving data for offline analysis. Inset: Photograph (highlighted with a yellow outline) showing four mice standing on the platform during the pre-start period.

To overcome limitations of traditional TST setups, we developed a custom-designed TST apparatus consisting of a recording chamber with a removable resting platform, an integrated webcam, a speaker, and an iMOSS-AS hardware module controlled through a Bonsai-RX workflow (Figures 1A–C).

The recording chamber is built from acrylic panels (Figure 1A) using a modified design based on a chamber described in a previous publication (Can et al., 2012), with detailed dimensions and assembly schematics are shown in Figure 1—Supplementary Figure 1 and Figure 1—Supplementary Table 1. The integrated removable resting platform offers several practical advantages over standard commercial chambers. It ensures synchronized trial starts and prevents the experimenter from accidentally blocking the camera during recording. Additionally, the design simplifies cable management for optogenetics and fiber photometry. While researchers may use commercially available recording chambers as an alternative, doing so often sacrifices these integrated benefits, leading to a less consistent and more cumbersome experimental workflow. This chamber, paired with a 30-fps camera and a white noise speaker, provides a complete solution for video-based TST. It generates the high-quality video data required for iMOSS-MV—our newly developed software for annotating mobility and immobility—ensuring a reliable and streamlined workflow from recording through to data analysis.

iMOSS-AS hardware module extends this design with a motion detection module (Figures 1B,C). With this add-on module, struggle-induced force fluctuations generated by mouse movements were translated into electrical signals using a strain-gauge load cell (Sparkfun, TAL221; 100 g capacity) integrated into the suspension bar. The analog output from the load cell was conditioned by an amplifier (Sparkfun, HX711) and digitized via an Arduino Uno running customized firmware built on the HX711-ADC library (Figures 1B,C and Figure 1—Supplementary Table 1). A calibration coefficient was determined once during hardware setup using a standard weight (50 g), and this coefficient was embedded in the firmware to convert amplified load-cell signals into milligram-scale force units, consistent with standard strain-gauge-based digital scales. The calibrated force measurements were streamed into a dedicated Bonsai-RX workflow (Figures 1B,D), which logged the signal throughout each session for subsequent conversion to grams during downstream analysis in the iMOSS-AS software. This configuration captures mechanical and visual signals in parallel, providing enhanced temporal precision and behavioral resolution.

Using this configuration, up to four mice can be placed simultaneously on the resting platform, with their tails attached to the hanging bars using adhesive tape. The 6-min TST session will be initiated when the resting platform is abruptly removed via manual actuation, during which the struggle signals and video data are synchronously recorded (Figure 1D).

### iMOSS-MV: a frame-accurate, video-based manual scoring software

**FIGURE 2:**
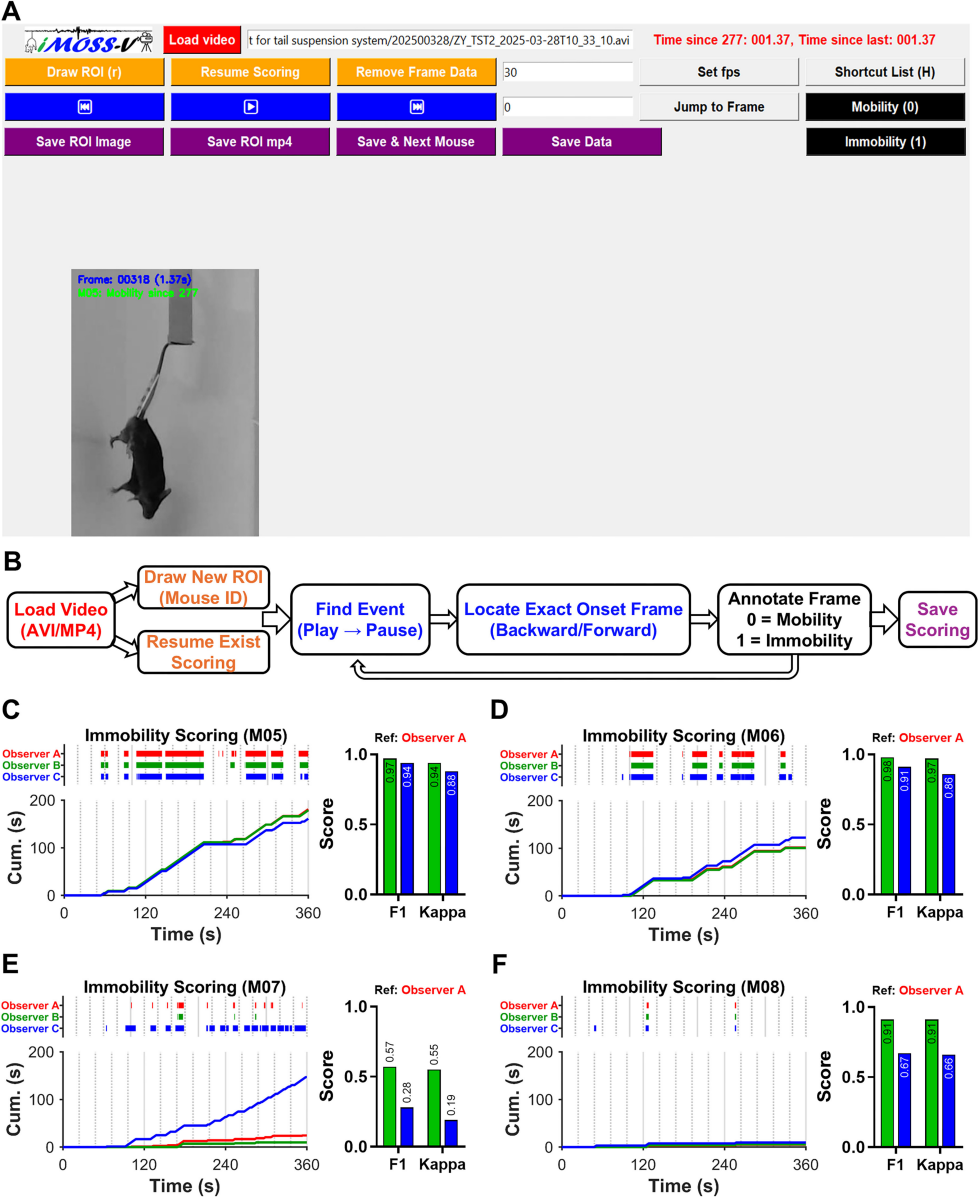
iMOSS-MV software for frame-accurate manual video scoring of immobility and mobility. **(A)** User interface illustrating video loading (red), region of interest (ROI) selection, and frame-by-frame scoring controls (blue). The software also allows users to resume, edit, and export results to Excel. **(B)** Schematic overview of the iMOSS-MV workflow for frame-accurate manual immobility annotation. **(C–F)** Raster plots (top left) along with the cumulative immobility time traces comparison (bottom left) during a 30-min training session in four mice (**C,D**: saline-treated; **E,F**: imipramine-treated), scored by 3 different observers. The bar graphs of F1-scores and Cohen’s Kappa coefficients (right) showing the inter-rater reliability when compared to observer A.

To improve the frame-level accuracy and reproducibility of conventional manual scoring methods, we developed iMOSS-MV, a Python-based interactive platform for binary behavioral annotation (Figure 2A). The iMOSS-MV interface offers a streamlined workflow for loading videos, defining or retrieving ROIs, and resuming incomplete annotations (Figure 2B). Real-time frame-by-frame playback enables precise identification and annotation of event onset frames. Scoring progress can be saved at any stage and resumed seamlessly, ensuring annotation continuity. This design achieves temporal precision that is challenging to obtain with conventional manual scoring methods. Previously saved ROIs and annotations can also be reloaded for efficient review, modification, and training.

Inter-rater reliability was assessed using videos from four mice (M05–M06 from saline-treated group; M07–M08 from imipramine-treated group) independently scored by three observers. Observer A, the developer of iMOSS-MV, trained observers B and C (a postdoctoral trainee and a post-baccalaureate trainee, respectively) to classify mobility and immobility behaviors using the software. Both trainees received a single 30-min training session. Coherent levels of the raters were determined by two measures: *F1-scores*, the proportion of correctly predicted positive cases out of all the cases predicted as positive, and *Cohen’s Kappa coefficients*, agreement level between two raters. The scores of observer B were highly consistent with those of observer A across all mice: M05-M08 (Figures 2C–F), whereas observer C’s results were consistent with the other two observers for three mice (M05, M06 and M08) but diverged for the remaining one (M07). These findings indicate that iMOSS-MV supports reproducible, high-resolution manual scoring after minimal training, particularly in saline-treated animals. However, like other observer-based approaches, variability between observers can occur, most notably in imipramine-treated mice exhibiting reduced immobility durations, likely reflecting differences in prior research experience and potentially mitigable through additional training.

### iMOSS-AS: a sensor-based automated immobility scoring platform

**FIGURE 3:**
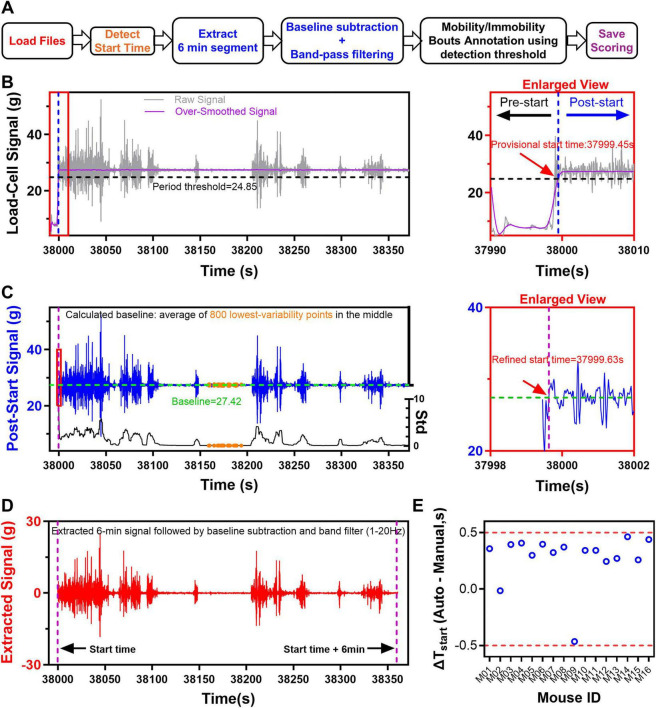
Development of sensor-based iMOSS-AS for signal extraction and immobility scoring. **(A)** Schematic overview of the iMOSS-AS workflow for automatic immobility annotation. **(B)** Representative plots illustrating the process of separating pre- and post-start periods. Left: the raw load-cell signal (gray) was heavily smoothed (magenta) to obtain a period threshold (black dashed line), which was then used to identify a provisional start time (blue dashed line)—defined as the earliest point after which the smoothed signal remained above the period threshold for at least 270 s. Right: enlarged view of the provisional start time separating pre- and post-start periods. **(C)** Refinement of the start time for the TST. The definitive start point (purple dashed line) was defined as the first time-point within the post-start period exceeding the baseline (green dashed line) calculated as the mean of 800 points with the smallest standard deviation (orange dots) in the middle third of the trace. An enlarged view of the refined start time is shown on the right. **(D)** The 6-min segment was extracted beginning at the definitive start time, followed by baseline subtraction and band-pass filtering (1–20 Hz). **(E)** Scatter plot showing difference between automatically and manually detected start times across 16 mice, with all values within 0.5 s.

iMOSS-AS software uses a two-step procedure to determine the start time of a session from recorded load-cell signals, defines the 360-s observation period, and establishes immobility detection threshold, which helps to distinguish immobility from mobility (Figure 3A). The first step involves identifying a provisional start time, which temporarily divides the recording into pre-start and post-start periods. This separation takes advantage of the distinct baseline load-cell signal levels (Figure 3B, right graph), with the mean signal of the post-start period (the mouse is suspended by its tail) greater than that of the pre-start period (the mouse stands on the platform, Figure 1E) due to its weight. To determine a provisional start time, raw load-cell signals are first heavy smoothed with a zero-phase, 100 data point moving-average linear digital filter (scipy.signal.filtfilt), implemented via forward-backward filtering (Figure 3B, left graph). The smoothed signals are then processed with the weighted formula (0.9 × the overall mean of over-smoothed signals + 0.1 × the smallest value of over-smoothed signals), to estimate a period threshold. Provisional start time is identified as the time point after which the smoothed signals remain greater for at least 270 s than the period threshold value (Figure 3B, right graph).

In the second step, iMOSS-AS estimates the baseline as the mean of the 800 lowest-variability points, defined by the smallest rolling standard deviation (Figure 3C, left graph), within the middle third of the post-start period. The refined start time is then defined as the first time point at which the post-start signal exceeds this baseline value (Figure 3C, right graph). A 360-s observation window is subsequently extracted beginning at the refined start time, followed by baseline subtraction and band-pass filtering (1–20 Hz) (Figure 3D).

This procedure was performed in 16 mice, and the iMOSS-AS package dramatically accelerates data analysis, processing results from all 16 mice in under 4 min, representing a substantial improvement over manual scoring, which requires more than 8 min per mouse. The automatically detected start times deviated by less than 0.5 s from manually annotated references, demonstrating high temporal accuracy (Figure 3E). Thus, this automatic start-time detection accurately defines observation periods and efficient batch processing of multiple recordings. Optionally, iMOSS-AS also provides users to manually define the start time by observing the raw signal or by referencing recorded videos.

**FIGURE 4:**
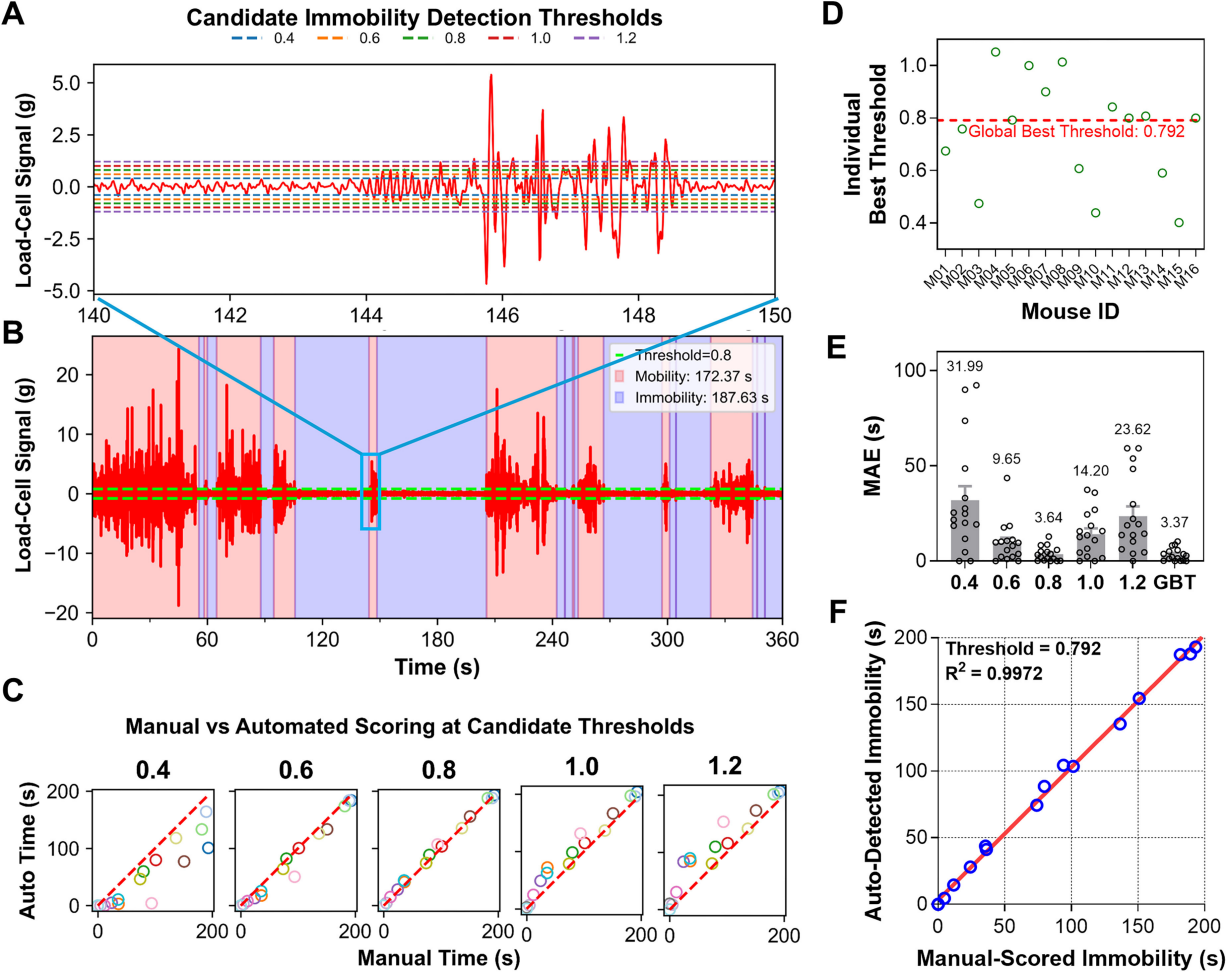
Machine learning-based optimization of immobility detection thresholds for iMOSS-AS. **(A)** Representative plots illustrating five candidate detection thresholds (0.4, 0.6, 0.8, 1.0, 1.2) determined based on the noise level of the extracted load-cell signal (red signal trace in **B**). **(B)** Example segmentation of mobility and immobility bouts is shown for the 0.8 threshold. **(C)** Scatter plots showing the relationship between iMOSS-MV-generated scores and iMOSS-AS-generated scores across the five candidate thresholds (red dashed line: *y* = *x*) (*n* = 16). **(D)** Distribution of optimal individual thresholds (green open circle) minimizing absolute deviations from manual annotations for each mouse, along with the global best threshold (GBT = 0.792, red dashed line) minimizing the average mean absolute error (MAE) across the cohort (*n* = 16). **(E)** MAE (mean ± SEM) produced by iMOSS-AS with the candidate thresholds and GBT. **(F)** Regression analysis between automated (iMOSS-AS) and manual (iMOSS-MV) measurements.

To quantify immobility, it is essential to determine an optimal signal-detection level that divides between mobility and immobility (i.e., threshold). Initially, iMOSS-AS estimated immobility counts for 16 mice using 5 visually inspected signal-detection thresholds (0.4, 0.6, 0.8, 1.0, and 1.2 g; Figures 4A,B). When compared to manual scoring (observer A using iMOSS-MV), each threshold produced systematic deviations, and the mean absolute error (MAE) exhibited a clear U-shaped relationship as a function of the detection threshold, with the minimum among the tested candidates occurring at 0.8 g (Figures 4C,E). This pattern suggested that the true optimum may lie between the tested values. Therefore, we used a Random Forest regressor (scikit-learn v1.5.1) to model this continuous relationship and estimate the threshold that minimizes the average MAE across the population. This approach reduces the discretization bias inherent in selecting from a limited set of candidate thresholds. By allowing interpolation within our tested range, it estimated a global best threshold (GBT = 0.792) that more precisely minimize prediction error (Figure 4D). We also calculated subject-specific optimal thresholds for validation purposes only. They are not used in the subsequent main analyses or in deriving the global threshold. Specifically, these individual thresholds allowed us to confirm that, although the optimal threshold varied across subjects, the global binary threshold (GBT) lay near the center of the observed distribution (Figure 4D). This supported the use of the GBT as a practical and unbiased threshold for group-level analysis.

To further verify the GBT, iMOSS-AS applied the GBT and calculated the total immobility time from these 16 mice, which produced the smallest average MEA among all 6 thresholds (3.37 ± 0.85 s, Figure 4E). A regression analysis also demonstrates its near-identical agreement with manual-scored times (slope = 0.996, *R*^2^ = 0.997, *p* < 0.001, Figure 4F).

To further demonstrate how well iMOSS-AS detects immobility over the course of the 360-s session, we compared the immobility counts of 16 mice between iMOSS-AS and observer A using iMOSS-MV. While the two methods depended on distinct procedures: sensor-based automatic vs. video-based manual, they produced nearly identical results (Figure 4—Supplementary Figure 1). Together, these results not only indicate the capability of iMOSS-AS but also confirm the reliability of iMOSS-MV.

### Performance comparison of iMOSS-AS with existing automated scoring systems

**FIGURE 5:**
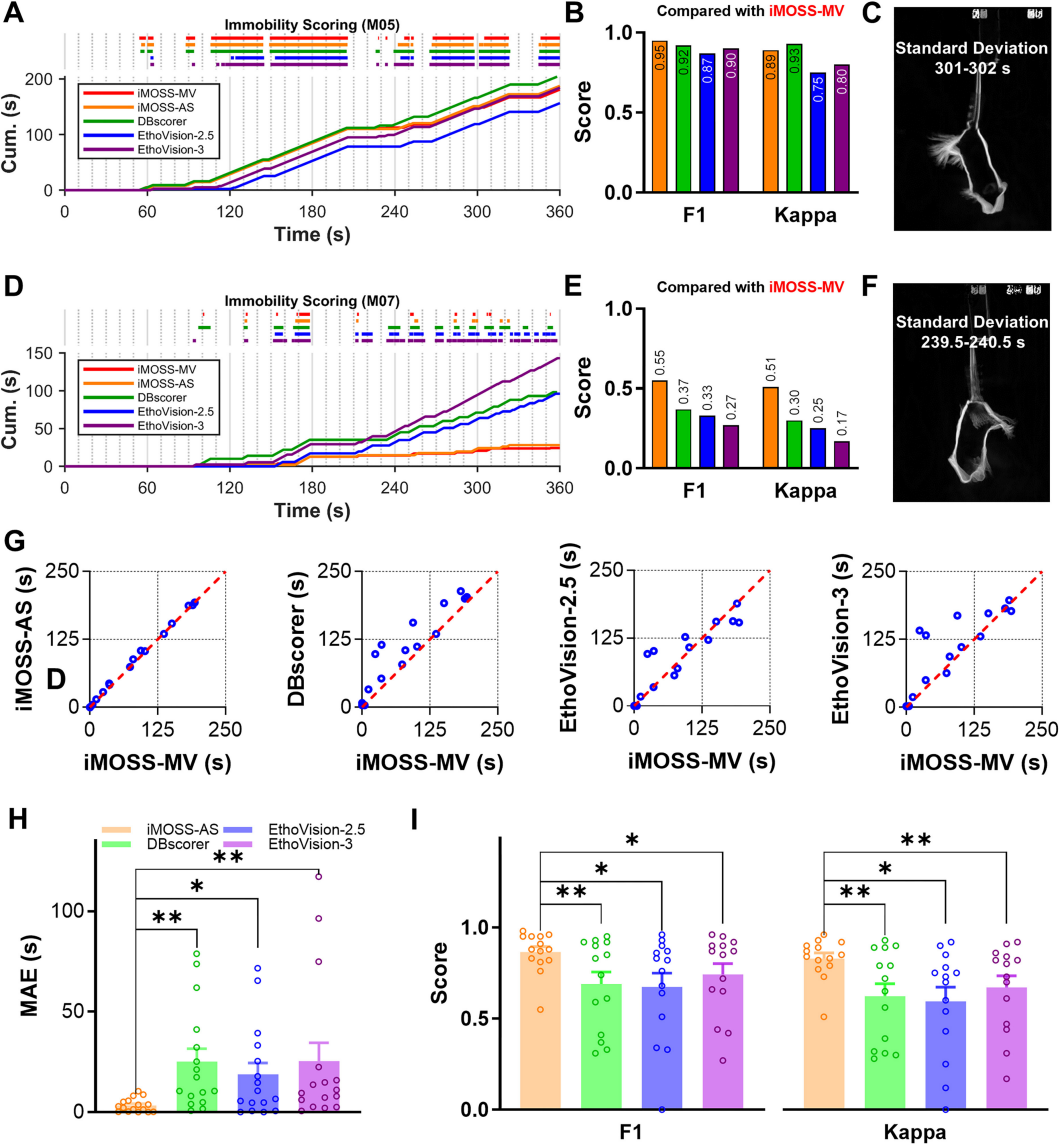
Comparison of iMOSS-MV, iMOSS-AS, and other automated scoring software. **(A,D)** Raster plots of immobility scoring and corresponding cumulative immobility times during the 6-min TST for mice M05 (saline-treated) and M07 (imipramine-treated) using iMOSS-MV (manual reference), iMOSS-AS, DBscorer, and EthoVision XT at thresholds of 2.5% (EthoVision-2.5) and 3% (EthoVision-3). **(B,E)** F1-scores and Cohen’s Kappa coefficients for the scoring of M05 and M07 using iMOSS-AS, DBscorer, EthoVision-2.5, and EthoVision-3, compared to manual reference. **(C,F)** Standard deviation plots illustrating motion contrast across a 1-second window (30 frames) for representative images of M05 and M07. **(G)** Scatter plots of immobility times scored between iMOSS-MV (x-axis) and automatic instruments: iMOSS-AS, DBscorer, EthoVision-2.5, or EthoVision-3 (y-axes). Each point represents one mouse (*n* = 16). The red dashed line represents the line of unity (y = x), illustrating the agreement between manual and automated scoring across methods. **(H)** MAEs ( ± SEM) generated by iMOSS-AS, DBscorer, EthoVision-2.5, and EthoVision-3. **(I)** Mean ( ± SEM) F1-scores and Cohen’s Kappa coefficients for iMOSS-AS, DBscorer, EthoVision-2.5, and EthoVision-3, in comparison to the manual reference, across all 16 mice. *p < 0.05, **p < 0.01.

Using manual scoring results obtained from iMOSS-MV as the reference standard, we compared the performance of iMOSS-AS with two widely used automated video-based scoring systems, DBscorer V2 (Nandi et al., 2021) and EthoVision^®^ XT (version 18; Noldus), to assess its accuracy in detecting immobility. Note that EthoVision enabled us to select activity detection levels, with a lower percentage level being more sensitive to movements and noises compared to a higher percentage level. Figures 5A–F show the example analyses from one mouse with high immobility (M05 from saline group) and another with low immobility (M07 from imipramine group), to capture a range of behavioral patterns during a 360-s session. All four tools—iMOSS-MV, iMOSS-AS, DBscorer, and EthoVision^®^ XT configured with 2.5% (EthoVision-2.5) and 3.0% (EthoVision-3) activity detection—produced comparable immobility estimates for M05 but diverged markedly for M07.

Frame-by-frame comparisons revealed that DBscorer and EthoVision often misclassified subtle movements as immobility (Figures 5A,D), particularly in the more active imipramine-treated mouse (Figures 5C,F). EthoVision-2.5 underestimated immobility in M05, whereas DBscorer and EthoVision with both configurations overestimated it in M07. These results suggest that the two publicly available systems are less sensitive to fine motor activity, leading to biased detection under conditions of subtle movement.

Across all 16 mice, immobility times scored by iMOSS-AS were highly consistent with manual annotations obtained using iMOSS-MV, whereas DBscorer and EthoVision showed larger deviations from the unity line (y = x) (Figure 5G). The MAE differed significantly among scoring methods [RM one-way ANOVA, *F*(3, 45) = 6.28, *p* < 0.05]. *Post hoc* Dunnett’s test indicated that iMOSS-AS yielded significantly lower MAE compared with DBscorer (*p* < 0.01), EthoVision-2.5 (*p* < 0.05), and EthoVision-3 (*p* < 0.01) (Figure 5H).

To compare performance quantitatively, we computed F1-scores and Cohen’s Kappa coefficients across all 16 mice, including representative examples from M05 (Figure 5B) and M07 (Figure 5E). In relation to the reference manual scores, iMOSS-AS consistently yielded the highest scores on both metrics, outperforming DBscorer and EthoVision (Figures 5B,E). Across the full dataset, both F1 and Kappa coefficients differed significantly among scoring methods [F1: *F*(2.2, 28.2) = 6.46, *p* < 0.01; Kappa: *F*(2.3, 29.5) = 7.61, *p* < 0.01]. *Post-hoc* Dunnett’s tests indicated that iMOSS-AS outperformed all other methods (*p* < 0.05) (Figure 5I). These findings indicate that iMOSS-AS provides more accurate and reliable detection of immobility than existing automated systems, particularly in conditions involving slight movements.

### Pharmacological validation of iMOSS with imipramine

To assess the sensitivity of iMOSS-AS in detecting pharmacologically induced changes in mobility and immobility, we compared its performance with manual scoring (iMOSS-MV) in mice treated with either saline (n = 8) or the antidepressant imipramine (15 mg/kg, i.p., 30 min before testing, n = 8), a standard positive control known to reduce immobility in the TST following acute administration. Treatment identities remained encoded until completion of all blinded analyses, as described previously.

**FIGURE 6:**
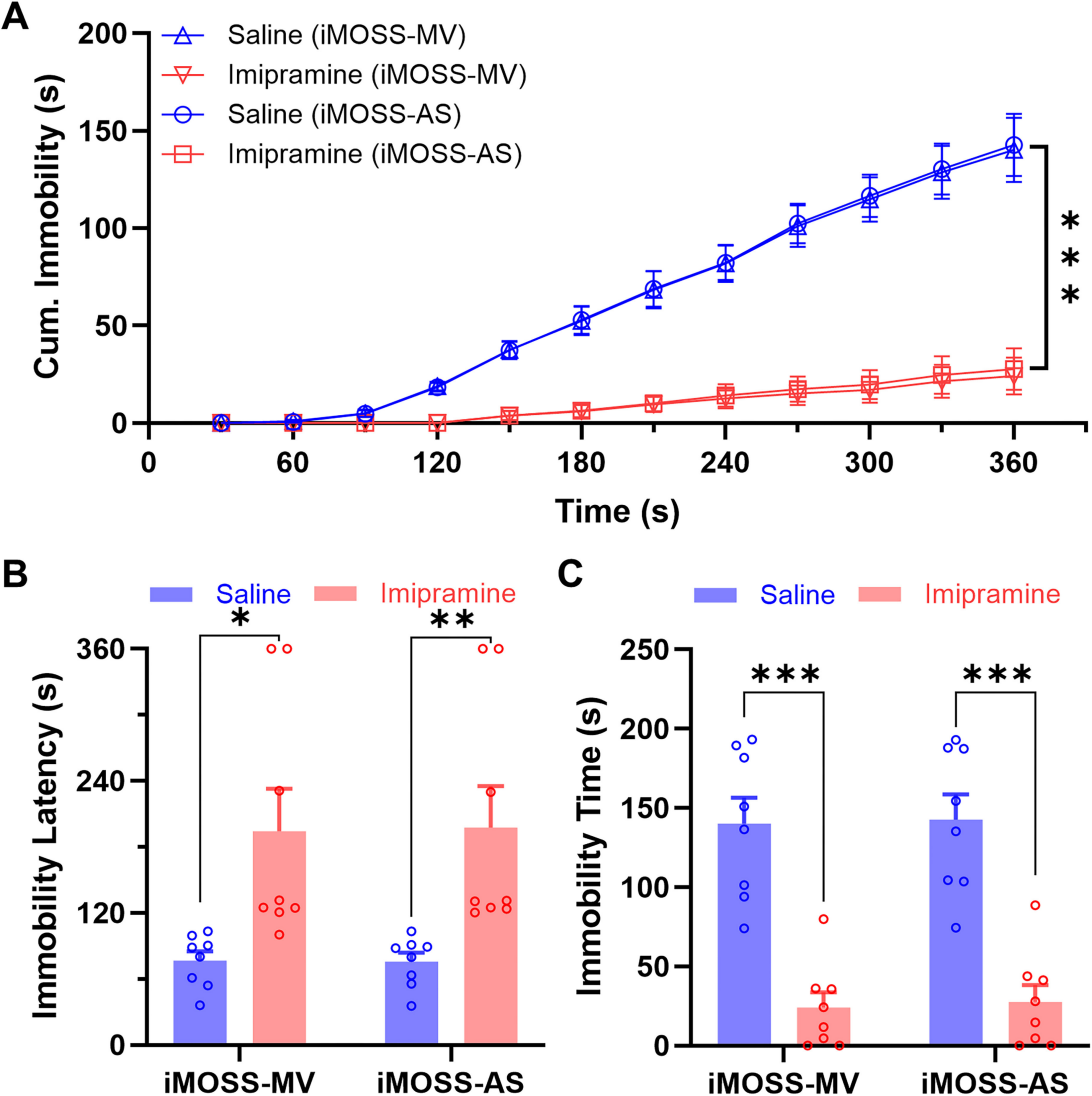
Pharmacological validation of iMOSS-AS reliability with imipramine. **(A)** Mean ( ± SEM) cumulative immobility scores across the 6-min test of saline- (*n* = 8) and imipramine-treated (*n* = 8) mice. **(B)** Mean ( ± SEM) immobility latency scored with iMOSS-MV and iMOSS-AS. **(C)** Mean ( ± SEM) immobility times scored with iMOSS-MV and iMOSS-AS. *p < 0.05, **p < 0.01, ***p < 0.001.

As expected, imipramine treatment significantly decreased immobility compared to saline controls (Figure 6A) A treatment x time x instrument ANOVA revealed main treatment effect [*F*(1, 14) = 46.86, *p* < 0.001], with no significant effect of instrument [*F*(0.1, 1.6) = 5.35, *p* = 0.087].

For immobility latency, two-way RM ANOVA revealed a significant increased latency in imipramine-treated mice compared with saline controls [Figure 6B; *F*(1, 14) = 9.42, *p* = 0.008], while no significant difference was detected between iMOSS-MV and iMOSS-AS [*F*(1, 14) = 0.39, *p* = 0.542] or for instrument x treatment interaction [*F*(1, 14) = 1.11, *p* = 0.310]. To further assess the temporal consistency between the two methods, we compared their immobility latencies on a subject-by-subject basis. The latency time from iMOSS-MV and iMOSS-AS were highly consistent, showing a near-perfect correlation (Figure 6—Supplementary Figure 2, *R*^2^ = 0.993). This strong temporal consistency is particularly noteworthy, as the model was trained using only the total immobility duration. This suggests that the model learned the underlying temporal dynamics of the behavior, not just a simple transformation of the total time. This successful generalization to a time-dependent feature provides strong evidence for the robustness of the underlying model and, by extension, the global best threshold derived from it.

For the total immobility time, two-way RM ANOVA showed significant main effects of scoring instrument [Figure 6C, *F*(1, 14) = 9.76, *p* = 0.007] and treatment [*F*(1, 14) = 36.58, *p* < 0.001], with no significant instrument x treatment interaction [*F*(1, 14) = 0.36, *p* = 0.557]. These findings indicate that although absolute immobility values differed slightly between scoring methods, both iMOSS-MV and iMOSS-AS reliably detected the treatment-induced reduction in immobility, confirming that iMOSS-AS can accurately quantify pharmacological modulation of stress-coping behavior with precision comparable to manual scoring, validating its use for high-throughput behavioral-pharmacological studies.

To demonstrate the overall sensitivity and robustness of our sensor system, we assessed its ability to detect a known pharmacological effect using several non-optimized thresholds. Total immobility times were compared between saline- and imipramine-treated mice using iMOSS-AS across all 5 candidate detection thresholds (0.4-1.2). Despite variations in absolute immobility durations, all thresholds consistently revealed a significant reduction in immobility following imipramine treatment (all *p* < 0.001; Figure 6—Supplementary Figure 1). This powerful result highlights the remarkable sensitivity of our measurement method. It suggests that while the ML-optimized threshold remains as the preferred approach—particularly for detecting subtle effects or for fine-grained temporal analysis—whereas empirically chosen thresholds may be adequate for straightforward confirmation of large effects and may reduce the labor required for such applications.

### Example integration of iMOSS with neural activity measurements

The high temporal resolution of iMOSS enables precise coordination of mobility and immobility responses with neural recordings, facilitating integrated analyses of brain–behavior relationships. iMOSS-AS samples behavioral signals at 80 Hz, while iMOSS-MV provides frame-by-frame manual scoring at 30 Hz, allowing both systems to be readily aligned with neural data streams.

**FIGURE 7:**
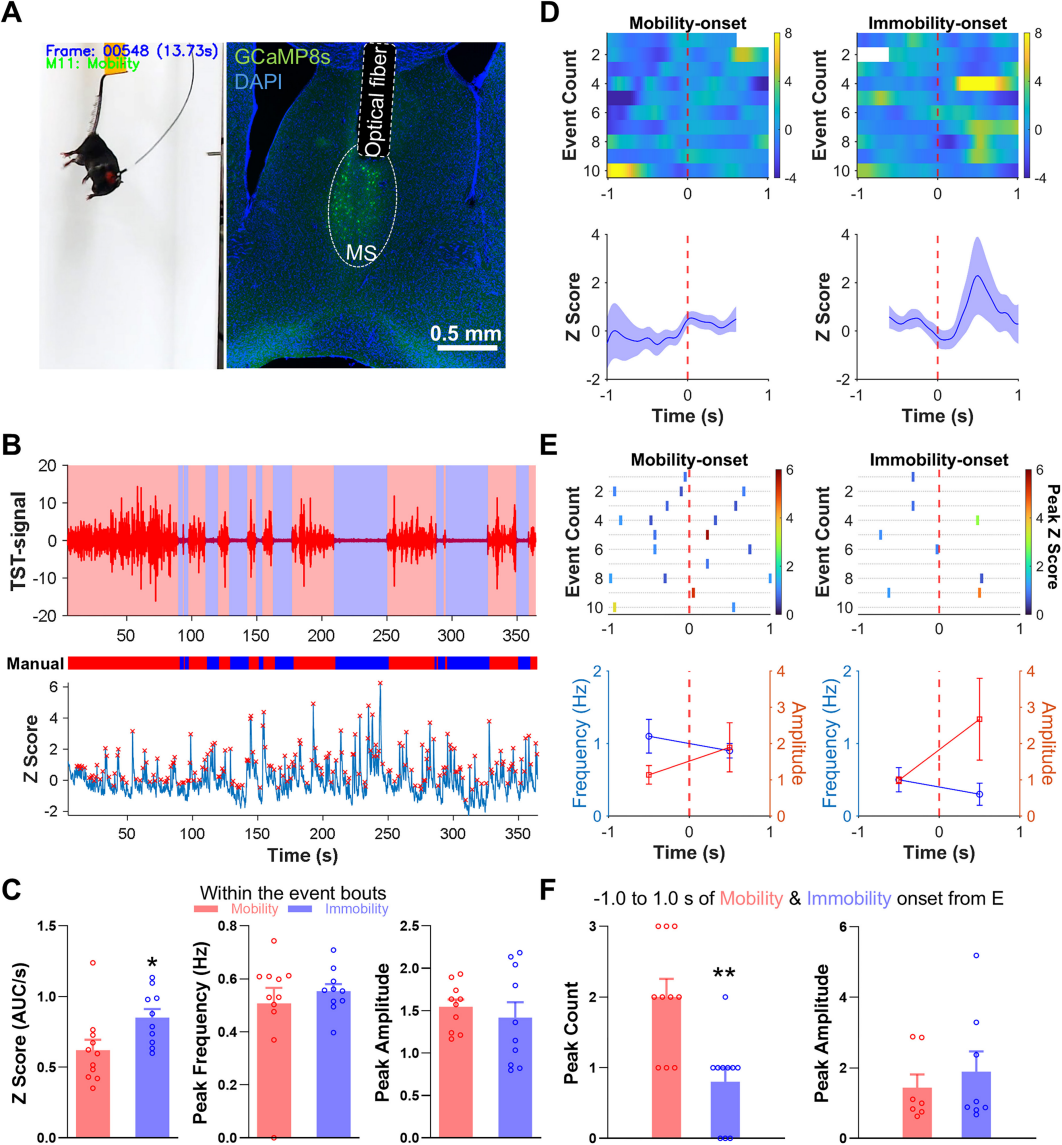
Behavioral scoring synchronization with neural activity in a single mouse. **(A)** Right: Representative image of a VGluT2-Cre mouse during the TST with patch cable coupled to the optical fiber cannula implanted in the medial septum (MS). Left: Green dots show GCaMP8s expression of MS VGluT2 neurons, while blue shows nuclear DAPI staining. **(B)** Load-cell signals detected by iMOSS-AS (top), manually scored mobility (red) and immobility (blue) bouts with iMOSS-MV (middle), and GCaMP8s signals recorded from MS vGluT2 neurons with calcium peaks (red crosses) (bottom). **(C)** Mean ( ± SEM) calcium Z score (left), peak frequency (middle) and amplitude (right) between immobility and mobility bouts. **(D)** Heatmap (top) and mean trace (bottom) of GCaMP8s signals within 1-s before and after the onset of mobility and immobility, scored by iMOSS-AS. The white regions inside the heatmap indicate masked time points where the aligned window exceeded the target event boundaries. Only event-pure segments were included in the analysis to avoid contamination from adjacent behavioral states, and averages were computed from valid samples only. **(E)** Raster plots (top) and line graphs (bottom) illustrating the frequency and amplitude of calcium peaks relative to mobility and immobility onsets. **(F)** Mean ( ± SEM) calcium peak count (left) and amplitude (right) within 1-s before and after the onset of immobility and mobility bouts. *p < 0.05, **p < 0.01.

To illustrate this capability, we conducted fiber-photometry recordings in a VGluT2-Cre mouse expressing GCaMP8s in the medial septum (MS) during a 360-s TST (Figure 7A). By aligning the struggle signal, particularly those scored by iMOSS-AS, with the calcium activity in MS VGluT2 neurons, we could examine the neural activity difference between mobility and immobility bouts (Figure 7B), by performing quantitative comparisons on the average Z score (AUC per second), calcium peak frequency, and peak amplitude across behavioral states (Figure 7C).

The MS has been implicated in defensive and related behaviors (Sheehan and Numan, 2000), and recent studies suggest that MS VGluT2 neurons increase activity at the onset of locomotor bouts (Fuhrmann et al., 2015). In this single-subject example, calcium activity in MS VGluT2 neurons closely tracked transitions between immobility and mobility states identified by iMOSS (Figures 7D–F). Heatmaps and averaged Z score traces showed that calcium signals slightly increased around mobility onset while decreased around immobility onset, followed by a rebound increase, reflecting dynamic modulation of MS activity by behavioral state (Figure 7D). Raster and line plots further quantified behavioral transition related changes in calcium peak frequency and amplitude (Figure 7E). Notably, the peak counts, but not the amplitudes, occurred within ± 1 s of mobility onset were significantly higher than those surrounding immobility onset [Figure 7F; *t*(18) = 3.67, *p* = 0.002].

These analyses conducted on a single subject demonstrate how iMOSS facilitates the synchronization of behavioral annotations with GCaMP signals, thereby providing a framework for the analysis of neural signals and TST behaviors.

## Discussion

This study introduces iMOSS-MV and iMOSS-AS, integrated open-source platforms for high-resolution quantification of immobility during the TST. iMOSS-MV enables frame-by-frame manual annotation with high temporal precision, while iMOSS-AS automatically analyzes load-cell signals with consistency, objectivity, and efficiency. Both systems provide temporally precise behavioral measurements suitable for direct synchronization with neural recordings, bridging a critical gap between behavioral and physiological data.

A central challenge in behavioral neuroscience is that, although manual observation is conceptually the most dependable method for scoring complex behaviors, its practical application is often limited. Video-based automated systems frequently overlook subtle movement nuances, whereas traditional manual scoring suffers from observer fatigue, inter-rater variability, and a lack of precise annotation tools, making it an imperfect “gold standard”. We contend that a scoring system is only as good as the ground truth against which it is validated. Improving this ground truth can be achieved either through hardware enhancements (e.g., multi-camera setup) or through more precise manual annotation tools. Given the constraints of our compact multi-animal chamber, we pursued a software-based solution and developed iMOSS-MV to establish a more rigorous, human-curated reference.

iMOSS-MV provides features that can partially mitigate limitations inherent to single-camera video recordings. In particular, its frame-by-frame interface enables users to detect subtle preparatory or proximal movements (e.g., shoulder or trunk contractions) that may precede overt limb movements, even when distal body parts are occluded from view. This capability can improve scoring reliability compared to conventional real-time or coarse video-based approaches. Because it relies on human interpretation, it is also inherently robust to variations in lighting and other artifacts that can confound automated systems. This makes iMOSS-MV a powerful standalone tool that is not only highly efficient, with a 6-min session typically scored in approximately 8 min, but is also flexible enough to be applied to other binary behaviors, such as freezing or grooming.

Critically, iMOSS-MV provides a practical approach to improving inter-rater and inter- laboratory reliability. Its ability to reload and review prior annotations creates an effective platform for observer training, allowing labs to build and maintain a consistent scoring standard. Our validation demonstrates the power of this approach: after just a single 30-min training session, new observers achieved a high consistency with an expert scorer, even when analyzing videos strategically selected for diverse immobility patten and drug treatments (Figures 2C–F). The single instance of divergence we observed underscores the system’s sensitivity for identifying where targeted training may be beneficial. These findings support iMOSS-MV as a robust solution for improving reproducibility. Future studies could further build upon this by creating curated training libraries from larger, more diverse video cohorts (e.g., different lighting conditions and mouse strains), which would further help minimize inter-rater and inter-laboratory variability.

Having established this highly accurate reference with iMOSS-MV, we then developed iMOSS-AS, a sensor-based platform comprising a hardware add-on module and automated scoring software, to serve as the “high-throughput workhorse.” The superior performance of iMOSS-AS is therefore not an incidental finding, but stems from two key advantages: First, as a sensor-based system, it measures force change directly and is therefore immune to the inherent limitations of video analysis. Second, it was optimized against the superior, human-curated ground truth from iMOSS-MV. Importantly, iMOSS-AS is not “trained” on observer annotations in the conventional machine-learning sense; rather, its performance is calibrated by adjusting a single parameter (the “Global Best Threshold”) to align immobility detection with human scoring. This relatively simple calibration step improves temporal correspondence with observer-defined immobility, but does not involve fitting complex features to a specific observer. This two-step validation ensures iMOSS-AS is exceptionally accurate, outperforming widely used video-based systems like DBscorer and EthoVision. In addition, iMOSS-AS offers substantially higher temporal resolution (80 Hz) than video-based systems, including iMOSS-MV and EthoVision (30 Hz, limited by camera frame rate) and DBscorer (which operates at an effective resolution of 1 Hz). This enhanced sampling rate allows iMOSS-AS to capture rapid behavioral transitions that may be missed by lower-frequency methods. To illustrate its efficiency, iMOSS-AS can analyze 16 TST sessions in under 4 min, a task that would take over 2 h to manually annotate using the video recordings. This rigorous development process also ensures the robust generalizability of the GBT used by iMOSS-AS. First, our system is designed to minimize variability arising from factors such as animal weight or baseline drift. The load cell signal is calibrated to standardized force units (mg) during setup, and the baseline is tared before each session. Importantly, the threshold is defined based on changes in force during movement rather than absolute body weight. As a result, the global best threshold (GBT) is expressed in standardized units and is largely independent of static load differences across animals or sessions. Second, the hardware characteristics—particularly the signal-to-noise ratio of the load cell and amplifier—are likely the primary determinants of generalizability across laboratories. In our system, built using specific SparkFun components, the noise level is consistently low (approximately ± 0.4 g; Figure 4A). Therefore, we expect the GBT to generalize well across setups that use similar hardware with comparable noise profiles. For experiments using different hardware, or to provide additional confidence, we supply a machine-learning-based script that allows users to verify or derive an experiment-specific threshold from their own data. This provides a straightforward and flexible approach to calibration across diverse experimental conditions. Finally, although the GBT was derived using total immobility time, it accurately predicts immobility time courses and latencies (Figures 5A,D; Figure 4—Supplementary Figure 1, and Figure 6—Supplementary Figure 2), suggesting that it captures the underlying behavioral dynamics and is therefore a robust parameter.

Together, the manual (iMOSS-MV) and automated (iMOSS-AS) systems were intentionally developed as complementary tools within a single workflow, rather than as redundant alternatives. iMOSS-MV is not used only to generate labels for training the automated system, although this is one of its key functions. It also serves as a reference method for validation, quality control, and analysis of existing video datasets, and it can be readily adopted by laboratories that already use camera-based TST setups without requiring additional hardware. By contrast, iMOSS-AS requires hardware integration but is designed for fully automated, objective, and high-throughput analysis. Once trained and validated against iMOSS-MV, it enables rapid and consistent scoring across large datasets, which is especially advantageous for large-scale studies where efficiency and reproducibility are critical. Thus, the rationale for maintaining both systems is that they address different experimental needs: iMOSS-MV is most useful for manual reference annotation, validation, and compatibility with existing video-based workflows, whereas iMOSS-AS is preferred for routine large-scale automated analysis. We therefore do not view one system as simply replacing the other; rather, they serve distinct but complementary roles.

Importantly, both iMOSS-MV and iMOSS-AS are readily compatible with concurrent neuronal recordings, enabling sub-second alignment between behavior and neural dynamics. As a proof-of-concept, we used iMOSS-AS data from one VGluT2-Cre mouse to demonstrate the system’s capability for precisely aligning neural recordings with behavioral transitions, a feature made possible by its high temporal resolution and accuracy. Looking forward, while the system is optimized for a binary immobility and mobility classification, the amplitude of the load-cell signal could be leveraged as a continuous ‘mobility score’ that reflects movement intensity. This analog signal could provide a richer variable for correlating with neural activity than a simple binary state, offering a promising direction for future analyses.

It is also important to consider the iMOSS platform in the context of modern deep-learning (DL)-based behavioral analysis pipelines, such as those built on DeepLabCut. These video-based approaches are highly powerful for extracting detailed kinematic, postural, and multi-feature behavioral information. However, they generally require substantial computational resources, technical expertise, and time for model training and validation, and their performance can be influenced by common video-related challenges such as lighting variability, camera angle, and body occlusion. In contrast, the sensor-based iMOSS-AS was designed to be accessible, robust, low-cost, and easy to implement, while also providing high temporal resolution. Because it directly measures force, it is not affected by video-related artifacts and offers a practical and precise approach for immobility quantification. We therefore view iMOSS not as a replacement for DL-based methods, but as a complementary solution optimized for studies in which simplicity, robustness, and direct measurement of immobility are the primary priorities.

In this study, our primary aim was methodological validation of the iMOSS platform rather than broad biological inference. Although the total sample size was modest (*n* = 16; 8 per group), the system showed high sensitivity, including clear detection of treatment effects (*p* < 0.001), which suggests low measurement variability and supports the precision of the platform. We also took steps to assess robustness at the analytical level. Specifically, the machine-learning framework used a strict animal-level train/test split and grouped cross-validation, so model performance was evaluated on unseen animals rather than on repeated observations from the same individuals. In addition, the dataset included behavioral variability across conditions, as saline- and imipramine-treated mice spanned a broad range of immobility values, providing an initial test of performance under differing behavioral states. Future studies including larger cohorts, additional treatment conditions, different mouse strains, and independent laboratory settings will be valuable for further establishing the generalizability of the system. In parallel, potential hardware refinements include a remotely actuated, Bonsai-RX-controlled resting platform and a custom PCB adapter to simplify the Arduino Uno’s connection with the load cell amplifiers.

The TST remains widely used, although its translational relevance to clinical depression is debated and ethical considerations surrounding stress-based assays have been discussed. Alternative, less aversive assays (e.g., sucrose preference, nest building, and social interaction tests) are available for studying depression-related phenotypes. In the present study, the TST was not intended to model depression *per se*, but to induce a transient state of acute psychological distress for probing underlying neural mechanisms. Compared to some alternatives (e.g., the forced swim test), it also avoids risks such as hypothermia and prolonged physical strain. In this context, the use of such paradigms should be guided by the 3Rs principles (replacement, reduction, and refinement). High-precision tools such as iMOSS can help maximize the information obtained from each experiment, supporting more efficient study design and potentially reducing the number of animals required. Nonetheless, careful monitoring and appropriate welfare considerations remain essential.

In summary, iMOSS-MV and iMOSS-AS offer versatile and validated open-source platforms, providing highly affordable and attractive choices for many research groups. They surpass existing video-based tools and facilitate precise alignment of behavioral and neural measurements. By enabling reproducible and high-resolution quantification of stress-coping behavior, iMOSS provides a powerful platform for advancing mechanistic understanding of how the brain regulates active and passive responses to inescapable stress.

## Data Availability

The datasets presented in this study can be found in online repositories. The names of the repository/repositories and accession number(s) can be found at: https://github.com/addy9908/iMOSS.

## References

[B1] Can A. Dao D. T. Terrillion C. E. Piantadosi S. C. Bhat S. Gould T. D. (2012). The tail suspension test. *J. Vis. Exp.* 59:e3769. 10.3791/3769 22315011 PMC3353516

[B2] Fuhrmann F. Justus D. Sosulina L. Kaneko H. Beutel T. Friedrichs D. et al. (2015). Locomotion, theta oscillations, and the speed-correlated firing of hippocampal neurons are controlled by a medial septal glutamatergic circuit. *Neuron* 86 1253–1264. 10.1016/j.neuron.2015.05.001 25982367

[B3] Juszczak G. R. Sliwa A. T. Wolak P. Tymosiak-Zielinska A. Lisowski P. Swiergiel A. H. (2006). The usage of video analysis system for detection of immobility in the tail suspension test in mice. *Pharmacol. Biochem. Behav.* 85 332–338. 10.1016/j.pbb.2006.08.016 17049370

[B4] Lopes G. A. Bonacchi N. Frazã£O J. O. Neto J. P. Atallah B. V. Soares S. et al. (2015). Bonsai: An event-based framework for processing and controlling data streams. *Front. Neuroinformatics* 9:7. 10.3389/fninf.2015.00007 25904861 PMC4389726

[B5] Meng X. Xia Y. Liu M. Ning Y. Li H. Liu L. et al. (2024). A deep-learning-based threshold-free method for automated analysis of rodent behavior in the forced swim test and tail suspension test. *J. Neurosci. Methods* 409:110212. 10.1016/j.jneumeth.2024.110212 38960331

[B6] Nandi A. Virmani G. Barve A. Marathe S. (2021). DBscorer: An open-source software for automated accurate analysis of rodent behavior in forced swim test and tail suspension test. *eNeuro* 8:ENEURO.0305-21. 10.1523/ENEURO.0305-21.2021 34625460 PMC8570685

[B7] Sheehan T. Numan M. (2000). “The septal region and social behavior,” in *The Behavioral Neuroscience of the Septal Region*, ed. Numan R. (New York: Springer), 175–209. 10.1007/978-1-4612-1302-4_8

[B8] Steru L. Chermat R. Thierry B. Mico J. A. Lenegre A. Steru M. et al. (1987). The automated tail suspension test: A computerized device which differentiates psychotropic drugs. *Prog. Neuropsychopharmacol. Biol. Psychiatry* 11 659–671. 10.1016/0278-5846(87)90002-9 2894041

