# Supplementary material for: iMOSS: an integrated open-source tail suspension test platform for high-resolution immobility scoring and synchronization with neural activity
Source: Front Behav Neurosci. 2026 May 8;20:1819512. doi: 10.3389/fnbeh.2026.1819512 (PMC13194518; doi:10.3389/fnbeh.2026.1819512)
Supplement: Supplementary file 1 [file Data_Sheet_1.pdf]

Supplementary Materials

iMOSS: An integrated open-source tail suspension test platform for high-resolution immobility scoring and synchronization with neural activity

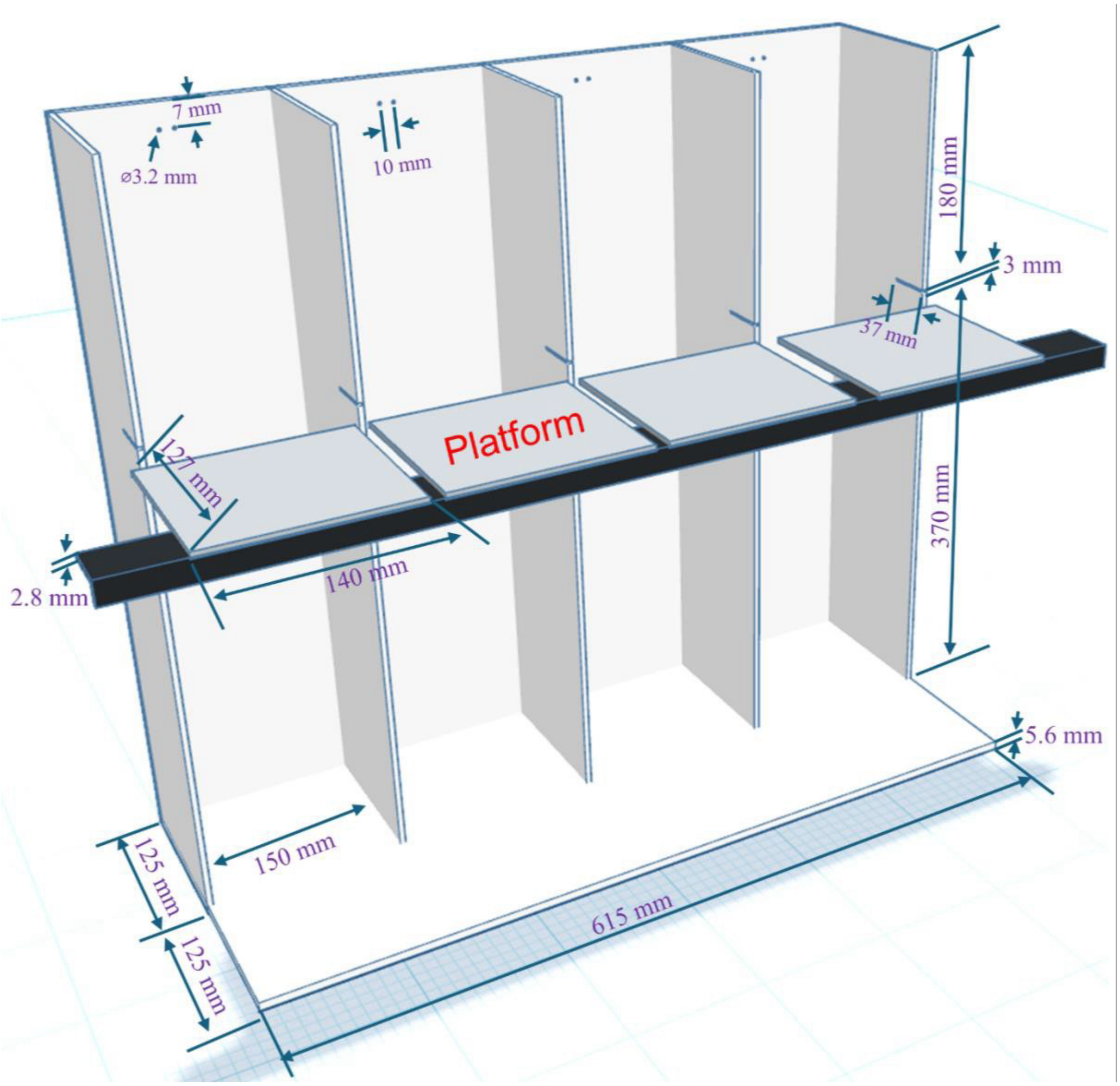

Figure 1 - figure supplement 1. Dimensions of the TST chamber.

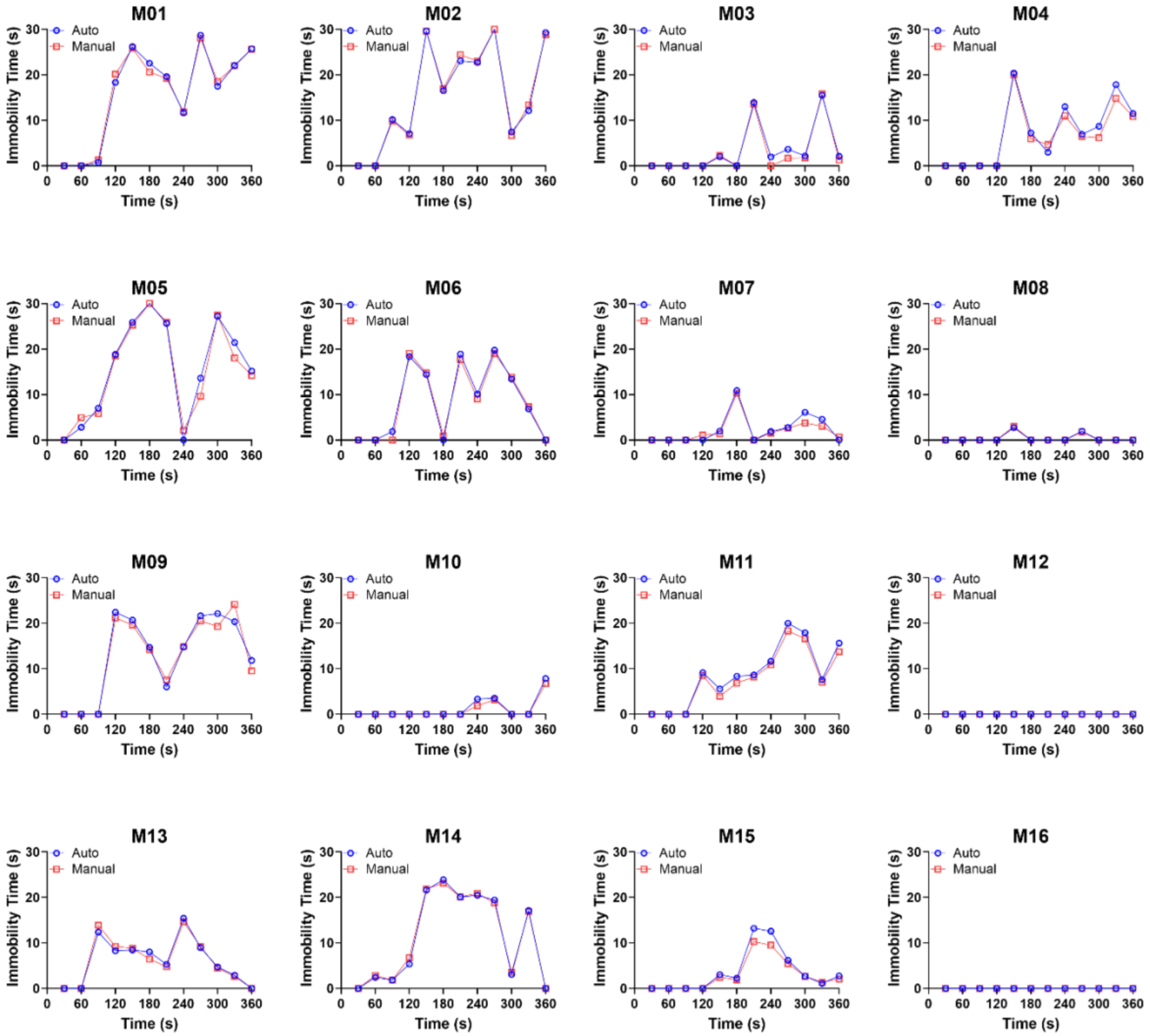

Figure 4 - figure supplement 1. Time-binned comparison of manual and automated immobility scoring. Line plots of immobility times in 30-s bins over a 6-min TST detected by observer A with iMOSS-MV and by iMOSS-AS for 16 mice.

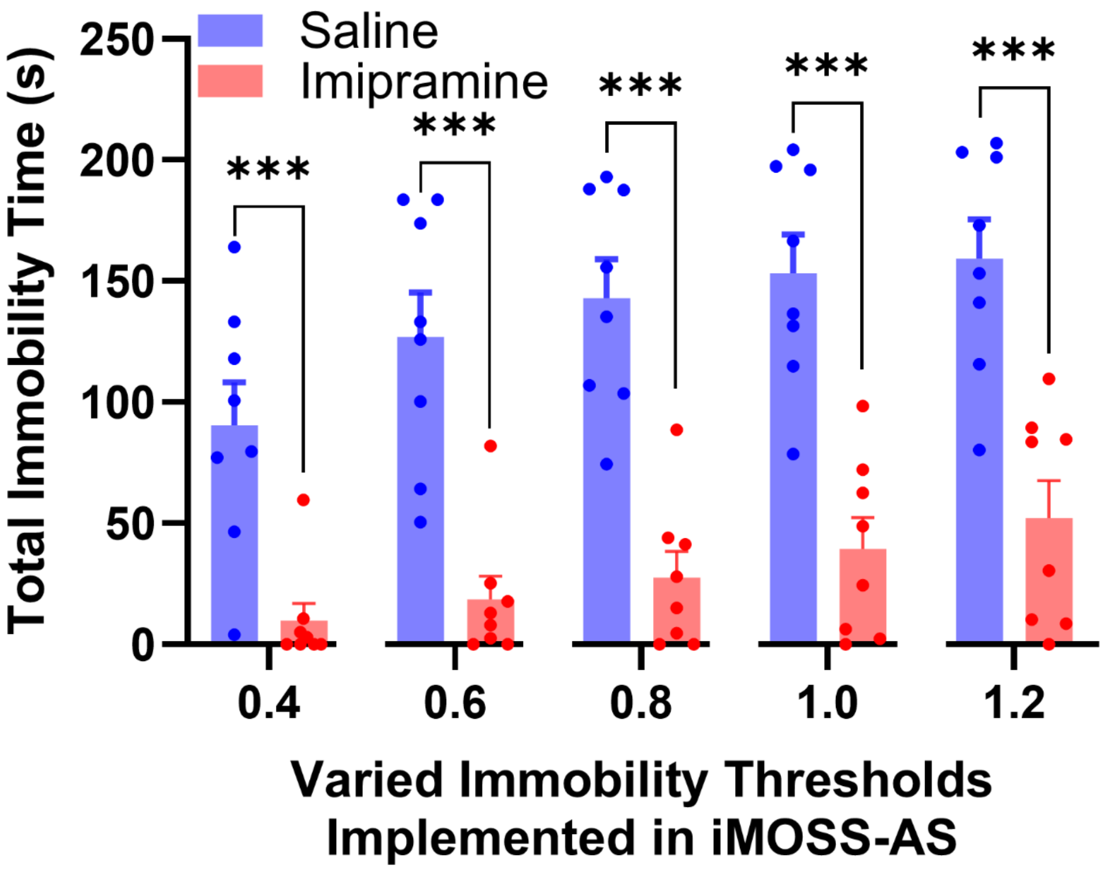

Figure 6 - figure supplement 1. Cumulative immobility times affected by mobility thresholds between saline- and imipramine-treatments. Total immobility times for saline-and imipramine-treated mice (n = 8 per group) were quantified using iMOSS-AS with all 5 candidate immobility detection thresholds (0.4–1.2), all of which showed a significant reduction in immobility after imipramine treatment. Unpaired 2-tail t-tests, *$p < 0.001$.

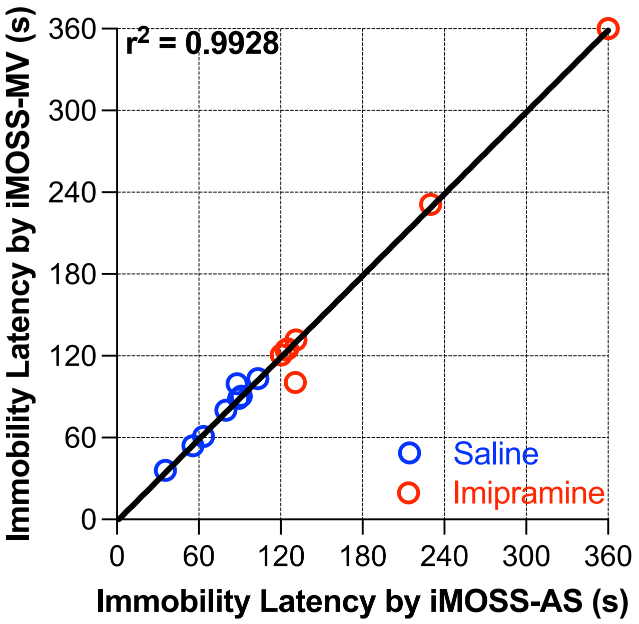

Figure 6 - figure supplement 2. The subject-by-subject scatter plot for immobility latency detected by iMOSS-MV and iMOSS-AS for mice treated with saline or imipramine.

Figure 1 - table supplement 1: Material list for iMOSS system

Category	Item Description	Unit Price	Qt	Source
Chamber materials	White Opaque Acrylic Sheet: 5.5 x 5 x 1/8" (Platform)	$3.60	4	Canalplastic.com
	White Opaque Acrylic Sheet: 22 x 5 x 1/8" (Side walls)	$7.20	5	Canalplastic.com
	White Opaque Acrylic Sheet: 24 x 10 x 1/4" (Base)	$24.00	1	Canalplastic.com
	White Opaque Acrylic Sheet: 24 x 22 x 1/4" (Back wall)	$48.00	1	Canalplastic.com
	16U Vertical Server Rack Rail	$27.98	1	Amazon.com
Electric parts	Logitech C922 Pro Webcam	$76.92	1	Amazon.com
	Mini Load Cell - 100g	$9.95	4	Sparkfun.com
	Load Cell Amplifier - HX711	$10.95	4	Sparkfun.com
	Arduino Uno R3	$23.46	1	Digikey.com
3D printed parts	Load cell holder: base		4	github.com/addy9908/iMOSS
	Load cell holder: top		4	github.com/addy9908/iMOSS
	Load cell holder: spacer		8	github.com/addy9908/iMOSS

Statistical Table

Figure	Method	Factor Name	Value	P Value
Fig. 4F	Simple linear regression	Slope non-zero	*F* (1, 14) = 4917	< 0.001
Fig. 5H	One-way<br>RM<br>ANOVA<br>Dunnett's post hoc test	Instrument (within)<br>iMOSS-AS vs. DBscorer<br>iMOSS-AS vs. EthoVision-2.5<br>iMOSS-AS vs. EthoVision-3	*F* (3, 45) = 6.28<br>*q* (45) = 3.74<br>*q* (45) = 2.65<br>*q* (45) = 3.77	= 0.001<br>= 0.001<br>= 0.029<br>= 0.001
Fig. 5I F1	One-way<br>RM<br>ANOVA<br>Dunnett's post hoc test	Instrument (within)<br>iMOSS-AS vs. DBscorer<br>iMOSS-AS vs. EthoVision-2.5<br>iMOSS-AS vs. EthoVision-3	*F* (2.2, 28.2) = 6.46<br>*q* (13) = 3.63<br>*q* (13) = 2.99<br>*q* (13) = 3.31	= 0.004<br>= 0.008<br>= 0.027<br>= 0.015
Fig. 5I Kappa	One-way<br>RM<br>ANOVA<br>Dunnett's post hoc test	Instrument (within)<br>iMOSS-AS vs. DBscorer<br>iMOSS-AS vs. EthoVision-2.5<br>iMOSS-AS vs. EthoVision-3	*F* (2.3, 29.5) = 7.61<br>*q* (13) = 4.00<br>*q* (13) = 3.50<br>*q* (13) = 3.91	= 0.002<br>= 0.004<br>= 0.010<br>= 0.005
Fig. 6A	Three-way ANOVA	Time (within)<br>Instrument (within)<br>Treatment (between)<br>Time X Instrument<br>Time X Treatment<br>Instrument X Treatment<br>Time X Instrument X Treatment	F (11, 154) = 75.18<br>F (0.1, 1.6) = 5.346<br>F (1, 14) = 46.86<br>F (1.7, 24.4) = 8.61<br>F (11.0, 154.0) = 35.96<br>F (0.1, 1.6) = 0.4354<br>F (1.7, 24.4) = 0.4999	< 0.001<br>= 0.087<br>< 0.001<br>= 0.002<br>< 0.001<br>= 0.196<br>= 0.588
Fig. 6B	Two-way<br>ANOVA<br><br>Šídák's post hoc test	Instrument (within)<br>Treatment (between)<br>Instrument X Treatment<br>Saline vs. Imipramine:<br>iMOSS-MV<br>iMOSS-AS	*F* (1,14) = 0.39<br>*F* (1,14) = 9.42<br>*F* (1,14) = 1.11<br><br>*t* (28) = 3.01<br>*t* (28) = 3.12	= 0.542<br>= 0.008<br>= 0.310<br><br>= 0.011<br>= 0.008
Fig. 6C	Two-way<br>ANOVA<br><br>Šídák's post hoc test	Instrument (within)<br>Treatment (between)<br>Instrument X Treatment<br>Saline vs. Imipramine:<br>iMOSS-MV<br>iMOSS-AS	*F* (1,14) = 9.76<br>*F* (1,14) = 36.58<br>*F* (1,14) = 0.36<br><br>*t* (28) = 6.07<br>*t* (28) = 6.01	= 0.007<br>< 0.001<br>= 0.557<br><br>< 0.001<br>< 0.001
Fig. 7C	Unpaired 2-tail t-test	Z-score<br>Frequency<br>Amplitude	*t* (19) = 2.38<br>*t* (19) = 0.70<br>*t* (18) = 0.64	= 0.028<br>= 0.495<br>= 0.533
Fig. 7F	Unpaired 2-tail t-test	Peak Count<br>Amplitude	*t* (18) = 3.67<br>*t* (15) = 0.39	= 0.002<br>= 0.706
